# Follicular fluid levels of vascular endothelial growth factor and early corpus luteum function during assisted reproductive technology cycles

**DOI:** 10.1186/1743-1050-2-13

**Published:** 2005-09-30

**Authors:** F Coppola, B Ferrari, L Barusi, V Caccavari, MC Salvarani, G Piantelli

**Affiliations:** 1 Center for Reproductive Medicine – Department of Obstetrics, Gynecology and Neonatology – University of Parma – 43100 Parma – Italy

## Abstract

**Background:**

The relation between vascular endothelial growth factor (VEGF) and early luteal function has rarely been proven in humans. The purpose of this study was to define the relation between follicular fluid concentrations of VEGF (FF VEGF) and early luteal function at the preimplantation stage during assisted reproductive technology (ART) cycles.

**Methods:**

71 women were divided into two groups, based on reproductive outcome: women who became pregnant after embryo transfer (ET) (n = 18, Group A) and non-pregnant women (n = 53, Group B). Serum progesterone (Se P) and inhibin A on ET day, and FF VEGF levels were measured in all women. Data were expressed as mean ± standard deviation. Statistical analysis was performed using Excel Office 98 for Student's t-test, linear regression test and chi-square test. A p value of < 0.05 was considered statistically significant.

**Results:**

The groups were comparable for age, ovarian reserve, number and quality of the oocytes retrieved and of the embryos obtained and transferred. FF VEGF levels were increased (4235 ± 1433 vs 3432 ± 1231 pg/ml), while Se P and inhibin A levels were significantly reduced (83.1 ± 34.1 vs 112.0 ± 58.8 ng/ml and 397.4 ± 223 vs 533.5 ± 283 pg/ml, respectively) in the non-pregnant group and were negatively correlated with FF VEGF (r = -0.482, p < 0.05; r = -0.468, p < 0.05) only in pregnant women.

**Conclusion:**

Much has to be learned about the regulation and role of VEGF during the early luteal phase. We advance the hypothesis that the existence of a negative correlation between FF VEGF/Se P and FF VEGF/inhibin A in pregnant women might indicate the existence of a normal VEGF-mediated paracrine response when Se P and inhibin A levels are decreased. Excess production of FF VEGF and the absence of a correlation between FF VEGF/Se P and FF VEGF/inhibin A in non-pregnant women may be a paracrine reaction to immature luteal vasculature, resulting in luteal dysfunction.

## Background

Several aspects of reproduction, from folliculogenesis to corpus luteum function, from embryo implantation to placental function, are related to angiogenesis [1-3]. The relation between vascular endothelial growth factor (VEGF) and luteal vascularization has been demonstrated in a large number of studies on animals [1-3], but it has rarely been proven in humans [4] and during assisted reproductive technology (ART) cycles [5]. This growth factor, which is produced by granulosa and thecal cells in response to FSH, LH and hCG production, and to hypoxia, is a potent angiogenic cytokine. It plays a prominent role in the development of an extensive, mature vascular network around the follicles and within the corpus luteum [4]. In a group of 13 women undergoing ART and regardless of reproductive outcome, Lee A. et al. [5] found that VEGF concentration in the follicular fluid (FF VEGF) at the time of oocyte retrieval (day 0) was positively correlated with serum (Se P) and follicular fluid (FF P) progesterone. This in turn indicates a correlation between FF VEGF and follicular luteinization at a very early stage, in a process that can be described as the first wave of angiogenesis. At 11 to 14 days after ET (day +11 to +13), the same authors [5] reported that the pregnant recipients of autologous fresh embryos had higher serum VEGF levels than both non-pregnant recipients of autologous fresh embryos and pregnant recipients of donor eggs. These data suggest that at onset of gestation, endogenous hCG produced by the embryo stimulates ovarian VEGF production, a process also known as the second wave of angiogenesis.

The purpose of this study was to define the relation between FF VEGF levels and early luteal function at the preimplantation stage – 3 days after oocyte retrieval (day +3), before endogenous hCG produced by the embryo stimulates ovarian VEGF production – and to verify whether dysfunction in VEGF production may interfere with luteal function during ART.

## Methods

### Patients

The study was performed on follicular fluid and blood samples routinely collected on oocyte pick-up day and 3  days after oocyte retrieval, respectively, in 71 infertile women referred for IVF-ET to the Center for Reproductive Medicine of the University of Parma Department of Obstetrics, Gynecology and Neonatology between September 2002 and March 2003. Inclusion criteria were: normogonadotropic women regularly menstruating with normal ovulatory function and normal corpus luteum processes, age under 40, and baseline FSH levels lower than 10 IU/L. Exclusion criteria were: genital conditions that might interfere with implantation, e.g. fibroma, hydrosalpinx, uterine malformations, polycystic ovary (PCO), endometriosis, and such complications as ovarian hyperstimulation syndrome (OHSS), chemical or clinical abortion. The patients were divided into two groups, based on reproductive outcome following ART: ongoing pregnancy after ET (Group A, n = 18) and non-pregnancy (Group B, n = 53). Permission for use of the follicular fluid and blood samples for this study was given by the University of Parma Ethics Committee. All patients gave their informed consent to IVF-ET.

### Stimulation protocol

All patients underwent controlled ovarian hyperstimulation (COH) following pituitary desensitization with a GnRH agonist (leuprolide 0.5 mg/day s.c. by Takeda Italia Farmaceutici, Rome, Italy) administered on day 21 of the cycle. The women were first monitored for inhibition of all ovarian activity and then stimulated with a standard dose of 225 IU of recombinant FSH (rFSH) (Gonal F 75 by Serono, Rome, Italy). The dose was later adjusted according to each patient's response. Ovarian response was monitored by serum estradiol (Se E_2_) assay and transvaginal sonography every other day. Finally, the patients received a dose of 10,000 IU of hCG (Profasi HP by Serono, Rome, Italy) when at least two follicles were > 16 mm and Se E_2 _levels were > 1000 pg/ml. The thickness of the endometrium was assessed by transvaginal sonography on the day of hCG administration.

### Oocyte retrieval and ET

Oocytes were retrieved for IVF 35 hours after hCG administration by transvaginal ultrasound-guided aspiration. The oocytes thus obtained were tested in the laboratory and classified into four maturation stages depending on the maturity of the oocyte-cumulus-corona complex. For the purpose of this study, oocytes were considered mature when they showed an extensive dispersal of the investing granulosa cells and an expanded cumulus and corona, while the zona pellucida was distinct and the ooplasm clear (Type 1). Oocytes of intermediate maturity manifested a slightly denser corona and a dispersed cumulus, and lacked a nuclear membrane (Type 2). Immature oocytes were surrounded by a compact corona and few cell layers in the cumulus (Type 3). Atretic or postmature oocytes displayed a dark, irregular ooplasm (Type 4). Embryos were classified according to the criteria proposed in 1994 by C. Staessen et al. [6]: Type 1, or excellent embryos (blastomers of equal size or, if not of equal size, without anucleate fragments); Type 2, or good embryos (blastomers of equal size or, if not of equal size, with up to 20% the volume of the embryo filled with anucleate fragments); and, Type 3, or fair embryos (anucleate fragments present in 20–50% the volume of the embryo). ET was performed 3 days after oocyte retrieval (110 hours after hCG administration). ET was considered difficult when there was blood on the catheter after transfer. The luteal phase was supported with 200 mg micronized progesterone (Esolut by Angelini S.p.A, Rome, Italy) administered daily (100 mg in the morning and 100 mg in the evening) by vaginal route, starting on the day of oocyte pick-up and continuing until the day of the pregnancy test.

Pregnancy was considered ongoing if serum levels of beta hCG (Se βhCG) at day 16 after oocyte pick-up were above 112 mIU/mL (10^th ^percentile of our trend curve) and confirmed by ultrasound at day 35 after oocyte pick-up.

### Follicular fluid and blood sampling and assays

The patients' blood samples were tested: i) on day 3 of the cycle, 3 to 6 months prior to ovarian stimulation (baseline FSH); ii) on day 6 of ovarian stimulation (Se LH); iii) on hCG day (Se E_2_); and, iv) on ET day (Se P and inhibin A).

Se E_2 _(pg/ml) and Se P (ng/ml) levels were determined in duplicate using a chemoluminescence kit (Medical System, Genoa, Italy). Se FSH (IU/L) and Se LH

(IU/L) were determined in duplicate by chemoluminescence using Coat-A-Count FSH and LH IRMA kits (manufactured by Euro/Diagnostic Products Corporation, Witney, Oxfordshire, UK).

Inhibin A measurement (pg/ml) was performed by solid-phase sandwich enzyme-linked immunosorbant assay (ELISA) using a DSL kit manufactured by Webster, Texas, USA, and distributed by Pantec, Turin, Italy.

Inhibin A reflects ovarian function and is a product not only of the fetoplacental unit but also of the corpus luteum in early pregnancy [7-11]. It would then represent a more reliable index of corpus luteum function than Se P in women concomitantly receiving vaginal micronized progesterone [7].

The follicular fluid pool from each patient was centrifuged at 900 g/min for 15 minutes to remove cellular and blood contamination and then kept at -70°C for the subsequent determination in duplicate of FF VEGF. The assay technique used in this study was: ELISA for total FF VEGF (pg/ml) (R&D System, Minneapolis, Minnesota, USA).

Intra- and interassay parameter variations were 4% and 4.2% for E_2_, 2.2% and 4% for FSH, 1% and 2.2% for LH, 6.2% and 6.7% for progesterone, 6.0% and 7.4% for inhibin A, respectively, and < 8% for VEGF.

### Statistical analysis

Data were expressed as mean (M) ± standard deviation (SD). Statistical analysis was performed using Excel Office 98 for Student's t-test, linear regression test and chi-square test. A p value of < 0.05 was considered statistically significant.

## Results

The characteristics of the patients under study – number, age, ovarian reserve, as well as Se LH levels on day 6 of ovarian stimulation (+6), and Se E_2 _levels on hCG day – are reported in Table 1.

**Table 1:** Patient population characteristics.

	Group A	Group B	p
Cases (no.)	18	53	
Age (M ± SD)	33.9 ± 3.6	34.0 ± 3.13	n.s.
Baseline Se FSH (IU/L) (M ± SD)	7.1 ± 2.4	6.8 ± 2.5	n.s.
Se LH, day +6 (IU/L) (M ± SD)	1.2 ± 1.0	1.3 ± 1.1	n.s.
Se E_2_, hCG day (pg/ml) (M ± SD)	1401 ± 597	1467 ± 716	n.s.
rFSH (IU/pt) (M ± SD)	3098 ± 949	3562 ± 1256	n.s.

As no significant differences emerged between women who became pregnant after ET (Group A) and non-pregnant women (Group B), the two groups could be considered comparable in view to the examined parameters. A comparison of data for the IVF-ET procedure in the two groups (Table 2) did not show any differences in the number and quality of the oocytes retrieved, in the embryos obtained and transferred in utero and in difficult ET. Measurement of endometrial thickness did not differ in the two groups, either (Table 2).

**Table 2:** IVF-ET data. Values are given as M ± SD

	Group A	Group B	p
Total oocytes/pt	8.4 ± 4.0	8.1 ± 4.3	n.s.
Oocytes type 1,1–2/pt	7.9 ± 3.6	7.0 ± 4.2	n.s.
Total embryos/pt	4.2 ± 2.0	3.7 ± 1.8	n.s.
Embryo type 1/pt	2.6 ± 1.8	2.0 ± 1.9	n.s.
Endometrial thickness (mm)	11.1 ± 2.9	10.9 ± 2.7	n.s.
Embryos transferred/pt	3.1 ± 0.8	3.0 ± 1.0	n.s.
Difficult ET (no./%)	1/18 5,5%	2/53 3,7%	n.s.

FF VEGF levels – an expression of the follicular paracrine environment – were significantly elevated (p = 0.039) in non-pregnant women (4235 pg/ml ± 1443) compared with women who became pregnant after ET (3432 pg/ml ± 1231) (Table 3).

**Table 3:** Follicular environment and luteal function. Values are given as M ± SD

	Group A	Group B	p
FF VEGF (pg/ml)	3432 ± 1231	4235 ± 1443	0.039
Se P (ng/ml)	112.0 ± 58.8	83.1 ± 34.1	0.013
Inhibin A (pg/ml)	533.5 ± 283	397.4 ± 223	0.042
FF VEGF vs Se P	r = -0.482 (a)	r = -0.178 (b)	(a) < 0.05; (b) n.s.
FF VEGF vs inhibin A	r = -0.468 (c)	r = -0.092 (d)	(c) < 0.05; (d) n.s.

(a) and (b) are correlation coefficients of FF VEGF to FF P

(c) and (d) are correlation coefficients of FF VEGF to inhibin A

**Figure 1:**
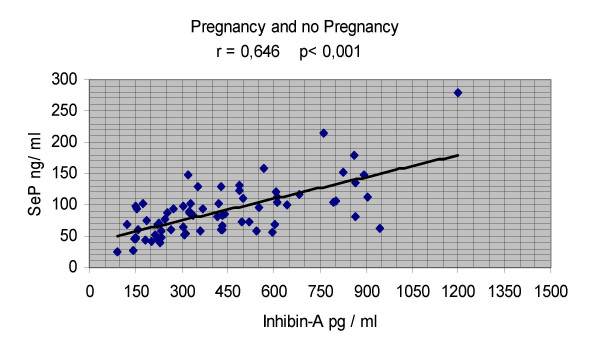
Correlation between Se P and inhibin A.

Se P and inhibin A levels measured in the early luteal phase were significantly (p = 0.013 and p = 0.04) elevated in Group A (112.0 ng/ml ± 58.8 and 533.5 pg/ml ± 283) versus Group B (83.1 ng/ml ± 34.1 and 397.4 pg/ml ± 223) (Table 3) and were negatively correlated with FF VEGF (r = -0.482, p < 0.05; r = -0.468, p < 0.05) only in Group A; no correlation (r = -0.179, p n.s.; r = -0.09, p n.s.) was found in Group B (Table 3). Despite the concomitant administration of vaginal micronized progesterone, Se P was well correlated (r = 0.646, p < 0.001) with inhibin A (Fig 1). This reduced the degree of inaccuracy that Se P might exhibit versus inhibin A in luteal function expression.

## Discussion

In our study, the two patient groups were comparable, not only for clinical characteristics, but also for the number and quality of the oocytes retrieved and of the embryos obtained and transferred in each group. Se P and inhibin A levels were significantly (p = 0.013 and p = 0.042) more elevated in pregnant women versus women who failed to conceive, while the angiogenic reaction was more marked (p = 0.03) in the latter. The cause of this excess angiogenic reaction is not yet clear. Under normal conditions, hCG administered to trigger ovulation induces the formation of a vascular network (first wave of angiogenesis) that helps ensure the correct development and functioning of the corpus luteum as a primary source of circulating progesterone [12] and inhibin A. In our study, the existence of a negative correlation (r = -0.482 and r = -0.468, p < 0.05) between FF VEGF/Se P and FF VEGF/inhibin A in pregnant women enabled us to demonstrate the existence of a normal VEGF-mediated paracrine reaction when Se P and inhibin A levels were decreased.

Beyond a given limit, as is the case with non-pregnant patients in whom there is no such correlation (r = -0.178 and r = -0.092, p n.s.), excess VEGF production is no longer enough to induce the formation of a mature vascular network in the corpus luteum through a paracrine mechanism and consequently to sustain adequate production of progesterone and inhibin A. This is also confirmed by studies on animals [13,1,14], revealing that luteal function can be impaired by anti-VEGF treatments down to a 50% reduction in Se P concentrations [2]. A large part of endothelial cells in the vascular network of the corpus luteum depend on VEGF support. Several variables, such as oxygen tension [15,16], insulin-like growth factor [3], aging [15], and poor response to ovarian stimulation [17], modulate the expression of this angiogenic factor. The pre-ovulatory follicle is a relatively large avascular multicellular structure [18]. Using mathematical formulas, it has been calculated that O_2 _content is potentially below the normal-range threshold (underoxygenation) [19], since the amount of O_2 _within mature follicles has been documented to range from less than 1% to about 5.5% [20]. Luteal expression of VEGF occurs primarily in specific perivascular cells, including arteriolar smooth muscle and capillary pericytes, and is regulated primarily by oxygen levels [12].

The reduced effect of exogenous hCG given to trigger ovulation on follicular luteinization in the women who failed to conceive and the consequent failure to achieve pregnancy in spite of supplemental progesterone administration in the luteal phase raises the question of whether exogenous hCG is capable of sustaining an adequate corpus luteum vascularization and whether progesterone can effectively substitute for luteal function. Our data seem to indicate that luteal function before ET is improved only in women who become pregnant. This might be due not only to improved corpus luteum vascularization, but also to a consequently adequate production of other local factors [21] of luteal origin (inhibin, oxitocin, growth factor, cytokines, prostaglandins and leucotrienes), which act in an autocrine or paracrine manner to modulate and perhaps mediate the development and function of the corpus luteum. Further studies are needed to investigate this aspect and lead to an in-depth understanding of the corpus luteum regulation mechanisms. The achievement of pregnancy following ART depends on a variety of factors (immunologic, genetic as well as endocrine, paracrine and vascular). In particular, getting to know with certainty why a woman fails to conceive is a crucial part of the process. Based on our study that evaluated only the maternal side prior to implantation in a selected sample of patients, we can hypothesize that suboptimal early luteal function – a condition related to immature vasculature – may play an important role in ART failure.

## Conclusion

Much has to be learned about the regulation and role of VEGF during the final stage of follicular development and the early luteal phase. We advance the hypothesis that the negative correlation between FF VEGF and the early luteal phase in pregnant women and elevated FF VEGF levels in non-pregnant women could be ascribed to a paracrine response compensating for perifollicular hypoxia and that luteal malfunction in women that fail to conceive could be the expression of an immature luteal vasculature. This mechanism is likely to occur when there is not enough angiogenic compensation and represents a clinical application of what has already been found in tests on non-human primates. However, further studies are needed to determine the role played by angiogenic factors or other substances, such as recombinant hCG, in controlling the growth and maturation of perifollicular and luteal vasculature, in order to find new therapeutic strategies that may help treat sterility by manipulating angiogenesis.

## Competing interests

The author(s) declare that they have no competing interests.

## Authors' contributions

FC have made substantial contributions to conception, design and interpretation of data. BF have made substantial contributions to interpretation of data. LB performed the statistical analysis. VC have made substantial contributions to acquisition of data.

MCS have made substantial contributions to acquisition of data. GP have been involved in revising the article critically.
